# Granulocyte Colony-Stimulating Factor Improves Endothelial Progenitor Cell-Mediated Neovascularization in Mice with Chronic Kidney Disease

**DOI:** 10.3390/pharmaceutics15102380

**Published:** 2023-09-25

**Authors:** Shao-Yu Tang, Yi-Chin Lee, Chien-Wei Tseng, Po-Hsun Huang, Ko-Lin Kuo, Der-Cherng Tarng

**Affiliations:** 1 Department of Medical Education, Taipei Tzu Chi Hospital, Buddhist Tzu Chi Medical Foundation, New Taipei City 23142, Taiwan; a0978916070@gmail.com; 2 Department of Physiology, National Yang Ming Chiao Tung University, Taipei 11221, Taiwan; yichinlee1022@gmail.com; 3 Department of Chinese Medicine, Taipei Tzu Chi Hospital, Buddhist Tzu Chi Medical Foundation, New Taipei City 23142, Taiwan; 107318138@gms.tcu.edu.tw; 4 School of Post-Baccalaureate Chinese Medicine, Tzu Chi University, Hualien 97004, Taiwan; 5 Institute of Clinical Medicine, School of Medicine, National Yang Ming Chiao Tung University, Taipei 11221, Taiwan; huangbs@vghtpe.gov.tw; 6 Cardiovascular Research Center, School of Medicine, National Yang Ming Chiao Tung, Taipei 11221, Taiwan; 7 Divisions of Cardiology, Department of Medicine, Taipei Veterans General Hospital, Taipei 11217, Taiwan; 8 Division of Nephrology, Taipei Tzu Chi Hospital, Buddhist Tzu Chi Medical Foundation, New Taipei City 23142, Taiwan; 9 School of Medicine, Tzu Chi University, Hualien 97004, Taiwan; 10 Division of Nephrology, Department of Medicine, Taipei Veterans General Hospital, Taipei 11217, Taiwan

**Keywords:** chronic kidney disease, granulocyte colony-stimulating factor, endothelial progenitor cell, neovascularization

## Abstract

Patients with chronic kidney disease (CKD) have a higher prevalence of peripheral arterial disease (PAD), and endothelial progenitor cells (EPCs) play a pivotal role. We examined the impact of granulocyte colony-stimulating factor (G-CSF) on EPC function in response to tissue ischemia. Eight-week-old male C57BL/6J male mice were divided into sham operation and subtotal nephrectomy (SNx) groups, received hindlimb ischemic operation after seven weeks, then randomly received G-CSF or PBS intervention for four weeks with weekly follow-ups. SNx mice had significantly reduced limb reperfusion, decreased plasma EPC mobilization, and impaired angiogenesis in ischemic hindlimbs compared to the control group. However, G-CSF increased IL-10 and reversed these adverse changes. Additionally, ischemia-associated protein expressions, including IL-10, phospho-STAT3, VEGF, and phospho-eNOS, were significantly downregulated in the ischemic hindlimbs of SNx mice versus control, but these trends were reversed by G-CSF. Furthermore, in cultured EPCs, G-CSF significantly attenuated the decrease in EPC function initiated by indoxyl sulfate through IL-10. Overall, we discovered that G-CSF can improve EPC angiogenic function through a hypoxia/IL-10 signaling cascade and impede neovascular growth in response to ischemia of SNx mice. Our results highlight G-CSF’s potential to restore angiogenesis in CKD patients with PAD via EPC-based methods.

## 1. Introduction

Peripheral arterial disease (PAD) is a disorder characterized by the occlusion of arteries outside of the heart, most commonly in the legs. Patients with chronic kidney disease (CKD) or end stage renal disease (ESRD) who have undergone dialysis are at enhanced risk of PAD occurrence compared to the general population [1,2]. The occurrence rate of PAD in patients with ESRD varies from 23% to 46%, which is significantly higher than the general population [3,4]. Therefore, healthcare providers face a distinct challenge when treating patients with both CKD and PAD, as the results of endovascular interventions for critical ischemic limbs are unfavorable [5]. Additionally, PAD is accompanied with a higher risk of all-cause death and cardiovascular morbidity, such as limb amputation [6,7]. Therefore, early detection and aggressive treatment of PAD in patients with CKD are essential to improve patient outcomes.

The risk factors for PAD in CKD patients are not yet completely understood. However, it is probable that both conventional risk factors and those associated with CKD play a role in the development of this condition. Patients with CKD frequently experience reduced capacity for the formation of collateral vessels in ischemic tissues, which may further increase their risk of PAD. To better understand this phenomenon, we conducted studies using an animal model of CKD by performing subtotal nephrectomy (SNx) and then mimicking PAD by inducing hindlimb ischemia (HI) through surgery [8,9]. Our previous studies showed that neovascularization in the ischemic hindlimbs of CKD mice was significantly worse than in sham mice [9]. SNx mice exhibited significantly increased plasma levels of indoxyl sulfate (IS), which is a protein-bound uremic toxin, as well as reduced reperfusion, decreased mobilization of plasma endothelial progenitor cells (EPCs), and hindered vascularization in ischemic hindlimbs compared to the sham-operated group. Additionally, we observed that ischemia-induced protein expressions, such as phospho-endothelial NO synthase (phospho-eNOS), hospho-STAT3 (p-STAT3), interleukin-10 (IL-10), and vascular endothelial growth factor (VEGF), were significantly downregulated in ischemic hindlimbs in SNx mice compared to control mice. Administration of AST-120, a special oral adsorbent of IS, successfully reversed these alterations. Through in vitro experiments, we further found that IS inhibits the HIF-1α and downstream VEGF molecular pathway and impairs EPC function [9].

Granulocyte colony-stimulating factor (G-CSF) is a cytokine that plays a crucial role in hematopoietic stem cell development and mobilization. Its therapeutic application involves the promotion of granulocyte production. For instance, recombinant human G-CSF is utilized in the treatment of patients with severe neutropenia during myelosuppressive therapy and bone marrow transplantation (BMT) [10]. Additionally, it is utilized during stem cell or BMT procedures to enhance the number of hematopoietic stem cells. However, recent research has suggested that G-CSF may offer other benefits, such as triggering a TAF9 to CASP3 signaling pathway to promote the preservation of retinal ganglion cells following ischemic optic neuropathy [11]. Administration of G-CSF has been found to result in significant reductions in interferon-γ and tumor necrosis factor-α, while also increasing levels of transforming growth factor-β and IL-10 [12]. Further studies reported that G-CSF treatment leads to the modulation of activated and suppressive G-neutrophils. Subsequently, it diminishes graft-versus-host disease associated with IL-10 and Treg [13]. Based on these findings, we explored whether G-CSF could enhance the impaired neovascularization that occurs in CKD. Specifically, our study aimed to investigate whether G-CSF could improve angiogenesis in ischemic hindlimbs by increasing EPC function in mice with remnant kidneys. We also examined the role of tissue hypoxia and the IL-10-associated downstream STAT3 and VEGF signaling pathway in vivo and investigated whether G-CSF increases the migration of cultured endothelial colony-forming cells (ECFCs) under hypoxic conditions.

## 2. Materials and Methods

### 2.1. Reagents and Antibodies

G-CSF was provided by UBI pharma Inc. (Taipei, Taiwan). Fluorescein isothiocyanate (FITC) anti-mouse Sca-1 and phycoerythrin (PE) anti-mouse Flk-1 antibodies were purchased from eBioscience (San Diego, CA, USA). Isotype-identical antibodies were obtained from Becton Dickinson (Franklin Lakes, NJ, USA). The antibodies listed below were purchased from Santa Cruz Biotechnology (Santa Cruz, CA, USA): rabbit anti-eNOS, anti-p-eNOS, mouse anti-VEGF, anti-α-SMA, and anti-β-actin. Rabbit antibodies for STAT3 and p-STAT3 were obtained from Cell Signaling Technology (Beverly, MA, USA). Mouse antibodies for IL-10 were purchased from Serotec (Oxford, UK). Rat anti-CD31 was obtained from BD Pharmingen (San Diego, CA, USA). Phenylmethylsulfonyl fluoride (PMSF) protease inhibitor, Histopaque-1077 (density: 1.077 g/mL), and 2,5-diphenyltetrazolium bromide (MTT) were obtained from Sigma-Aldrich (St. Louis, MO, USA). Endothelial growth medium (EGM-2 MV) and growth factors for EPC culture were obtained from Lonza (Morristown, NJ, USA).

### 2.2. Animals

All animal experimental procedures were conducted in compliance with Animal Research Reporting of In Vivo Experiments (ARRIVE) guidelines and followed the protocols approved by the Institutional Animal Care and Use Committee (IACUC) of National Yang Ming Chiao Tung University (Taipei, Taiwan); the IACUC approval number is 1050621. A total of 33 male C57BL/6JNarl mice were purchased from the National Laboratory Animal Center, National Science Council (Taipei, Taiwan). Experimental animals were accommodated in controlled environments with restricted access and maintained on a regular diet, adhering to a 12 h light and 12 h dark cycle.

### 2.3. Subtotal Nephrectomy

At 8 weeks old, 33 mice were allocated into three groups for the study: 7 mice for sham operation, 13 for SNx (subtotal nephrectomy) with no treatment, and 13 for SNx treated with G-CSF. An intraperitoneal anesthesia consisting of 100 mg/kg of ketamine and 10 mg/kg of xylazine was administered prior to surgery. The SNx procedure involved initially excising both poles of the right kidney, followed by the complete removal of the left kidney after a one-week interval, which was carried out according to a previously described method [8]. The sham-operated group of mice underwent the same surgery, but kidney ablation was not performed. After surgery, the mice were housed in individual cages and maintained until fully recovered, after which they were moved to their original cages.

### 2.4. Hindlimb Ischemia Surgery

Seven weeks after SNx, the induction of hindlimb ischemia (HI) surgery involved the removal of the right femoral artery, following the established method described earlier [9]. Prior to surgery, mice were anesthetized with a combination of 100 mg/kg ketamine and 10 mg/kg xylazine, followed by preoperative laser Doppler imaging. The skin and sarcolemma of the right thigh of mice were incised, and ligations were carried out on both the proximal and distal segments of the femoral artery, as well as the distal segment of the right saphenous artery. Subsequently, the arteries, along with their respective side branches, were carefully dissected and removed. Following the surgical procedure, laser Doppler imaging was employed to verify the establishment of the ischemia model. The G-CSF group of mice received weekly treatment with 690 μg/kg G-CSF by subcutaneous injection, and the SNx group was treated with phosphate-buffered saline (PBS) as a control.

### 2.5. Measurement of Renal Function and Indoxyl Sulfate

Blood samples were collected seven weeks after the SNx surgery and at the end of experiment to assess renal function and IS concentration. Serum was obtained from whole blood samples by centrifugation at 2500 rpm for 15 min, followed by careful collection of the supernatant. The resulting serum was then stored at −80 °C until measurement. The concentrations of blood urea nitrogen (BUN) and serum creatinine (Cre) were measured by an autoanalyzer (SpotChem EZ, SP-4430; Arkray, Edina, MN, USA). The concentration of plasma IS was determined using fluorescence-based high-performance liquid chromatography (HPLC). To prepare the samples, 50 mL of serum was combined with 150 mL of methanol to induce protein precipitation. After thorough mixing and subsequent centrifugation, the resulting supernatant (20 mL) was subjected to analysis. The separation of samples was accomplished using a Luna phenyl-hexyl column (150 × 4.6 mm, 5 mm; Phenomenex, Torrance, CA, USA). The mobile phase consisted of 10 mM phosphate buffer with 5 mM tetra-n-butyl ammonium hydrogen sulfate and acetonitrile (3:1), and the flow rate was 1.0 mL/min. For quantification, the excitation wavelength was 280 nm, and a 346 cutoff filter was used to measure emission.

### 2.6. Laser Doppler Imaging

A laser Doppler perfusion imager system (Moor Instruments Limited, Devon, UK) was utilized to measure the blood flow ratio between the ischemic limb (right) and the nonischemic limb (left) [14]. Following the administration of anesthesia (100 mg/kg ketamine and 10 mg/kg xylazine), the mouse was positioned with its ventral side up, legs spread apart, and hindlimb blood flow was sequentially monitored. Measurements were taken both before the hypoxic-ischemic surgery and on days 7, 14, 21, and 28 after surgery.

### 2.7. Flow Cytometry

Before and 2 days after HI surgery, a fluorescence-activated cell sorting FACSCalibur flow cytometer (Becton Dickinson, San Jose, CA, USA) was used to assess EPC mobilization [15]. A total of 100 mL of peripheral blood was incubated with FITC conjugated Sca-1 and PE conjugated Flk-1antibodies. Isotype-identical antibodies were used as controls. Following a 30 min incubation period, the cells were lysed using RBC lysis buffer for 10 min at room temperature. Subsequently, they were washed with phosphate-buffered saline and finally fixed in a 2% paraformaldehyde solution before analysis. Each analysis consisted of evaluating 80,000 events. Circulating EPCs were from the mononuclear cell population and were gated with a double positive for Sca-1 and Flk-1.

### 2.8. ELISA

Blood samples were collected before animals were sacrificed to assess cytokine concentration. The IL-10 concentration in the mice serum was determined using a commercially available enzyme-linked immunosorbent assay kit (R&D Systems, Minneapolis, MN, USA) according to the manufacturer’s instructions.

### 2.9. Histological Analysis

After the animals were sacrificed, the thigh muscles of their hindlimbs were dissected. Harvested muscle tissues were fixed with 4% paraformaldehyde for one day and then dehydrated and embedded in paraffin. Tissue blocks were cut into 5 mm sections, then deparaffinized and incubated for 10 min with 3% H_2_O_2_. After blocking with 1% BSA for 1 h, the samples were incubated with anti-CD31 or α–smooth muscle actin (α-SMA) antibodies overnight at 4 °C, and then incubated for 2 h with an HRP (horseradish peroxidase)-conjugated secondary antibody at 37 °C. The immunoreactions were visualized using a TE2000-U fluorescence microscope (Nikon, Melville, NY, USA). The number of CD31 or α-SMA positive cells were counted in 10 randomly selected high-power fields.

### 2.10. Western Blotting

Tissues were lysed with PBS containing 1% Triton X-100, 0.1% SDS, 0.5% sodium deoxycholate, 1 μg/mL leupeptin, 10 μg/mL aprotinin, 1 mM PMSF protease inhibitor, and phosphatase cocktail on ice. Following sonication, the tissue extracts were subjected to centrifugation at 12,000 times the force of gravity for 5 min at 4 °C. The resulting supernatants were collected as tissue lysates. To determine the protein concentrations, the Bradford protein-binding assay was employed. Aliquots containing 50 μg of protein from the lysates were separated using 8% SDS-PAGE and subsequently transferred onto an Immobilon^TM^-P membrane (Millipore, Bedford, MA, USA). After blocking with 5% skim milk for a duration of 1 h, the membranes were sequentially incubated with primary antibodies and then with secondary antibodies. Protein bands were detected by using an enhanced chemiluminescence kit (PerkinElmer, Boston, MA, USA) and quantified with ImageQuant 5.2 software (GE Healthcare Bio-Sciences, Pittsburgh, PA, USA).

### 2.11. Human EPC Isolation and Cultivation

The study about human EPCs was approved by the Ethics Committee of the Taipei Veterans General Hospital (Taipei, Taiwan) with IRB number 2016-08-002AC. Informed written consent was obtained from the participants. Human EPCs were isolated from human peripheral blood and expanded in culture as described previously [16]. Briefly, peripheral blood samples (20 mL) were obtained from healthy young adult volunteers, and total mononuclear cells were isolated by density gradient centrifugation with a Histopaque-1077. After being centrifuged at 1900 rpm for 30 min, a total of 5 million mononuclear cells were seeded onto fibronectin-coated 6-well plates in 2 mL of EGM-2 MV medium with supplements. After 4 days of incubation, the medium was replaced, and nonadherent cells were discarded, leaving behind elongated early EPCs with spindle-like shapes that had attached to the plate. Some of these early EPCs were allowed to further develop into ECFCs, which typically emerged within 2 to 4 weeks of initiating the mononuclear cell culture. The ECFCs exhibited a cobblestone morphology and displayed the characteristic monolayer growth pattern observed in mature endothelial cells when reaching confluence. These cells were identified as adherent cells that were positive for surface markers specific to endothelial cells and hematopoietic stem cells, including acetylated low-density lipoprotein (Dil-AcLDL), eNOS, Von Willebrand factor (vWF), CD31, CD34, VE-cadherin, and kinase insert domain receptor (KDR), as described previously [17]. The fluorescent images were recorded under a laser scanning confocal microscope. The ECFCs were collected and used for all the assays in this study.

### 2.12. MTT Assay

The cytotoxicity of G-CSF on ECFCs was assessed using a MTT assay. Isolated human ECFCs (2 × 10^4^) were placed in 96-well plates and then stimulated with G-CSF (45, 450, 4500, and 45,000 pg/mL) for 24 h at 37 °C. We used a normal culture medium without G-CSF as the control, which produced a 100% survival rate. The cell viability of ECFCs was determined by measuring absorbance at 540 nm and is reported relative to the control.

### 2.13. ECFC Migration and Tube Formation Assays

To assess the migratory capability of ECFCs, a modified Boyden chamber assay (Costar Transwell; Corning, Corning, NY, USA) was employed [18]. Isolated human ECFCs (4 × 10^4^) were added in the upper chambers of trans-well plates (24-well) with polycarbonate membrane (8 mm pores). After 24 h of incubation, washed membranes were stained with Hematoxylin for 5 min, and then were mounted on a slide to observe under an inverted light microscope. The extent of ECFC migration was determined by counting the migrated cells in six randomly selected microscopic fields at a magnification of 100×.

The in vitro tube formation of ECFCs was evaluated using an Angiogenesis Assay Kit (Millipore, Burlington, MA, USA) [14]. The 96-well microplates were coated with matrigel (40 μL/well) and allowed to polymerize for 30 min at 37 °C. ECFCs were added at 2 × 10^4^ cells/well into each matrigel-coated well in 100 μL of culture medium. After being cultured for 4 hrs, tube formation was examined under an inverted light microscope at a magnification of 100×. The number of branch points was assessed using image analysis for tube formation (Ibidi LLC, Verona, Germany) in four randomly chosen microscopic fields. EBM-2 served as the culture medium for both assays.

### 2.14. Statistical Analysis

The data presented represent the mean ± SEM values obtained from 4–13 independent experiments. The Mann–Whitney test was employed to compare two independent groups, while the Kruskal–Wallis test, followed by the Bonferroni post hoc test, was utilized for comparisons among multiple groups. Statistical analysis was conducted using SPSS software version 18.0 (IBM, Armonk, NY, USA). Statistical significance was determined at *p* < 0.05.

## 3. Results

### 3.1. Renal Function Decline and Increase in Plasma IS Levels after Subtotal Nephrectomy

**Figure 1:**
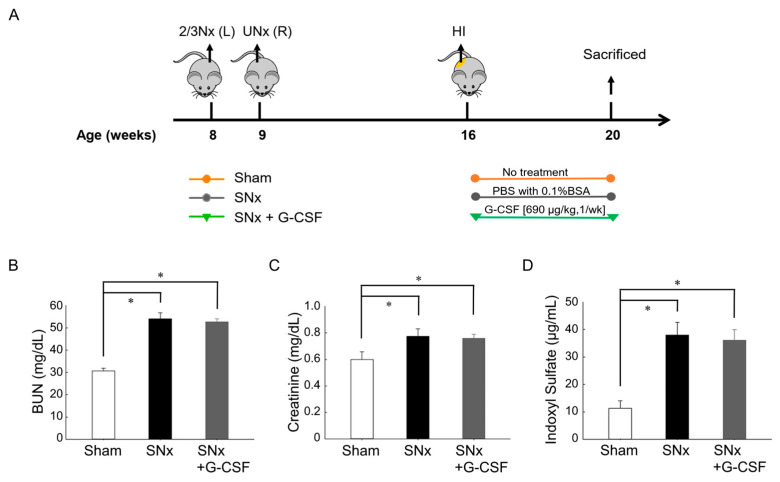
Study protocol and renal function and plasma indoxyl sulfate analysis in experiment mice. (**A**) The mice were categorized into three groups: the sham operation group (referred to as Sham), the subtotal nephrectomy group without any treatment (referred to as SNx), and the subtotal nephrectomy group treated with G-CSF (referred to as SNx + G-CSF). (**B**) Plasma BUN, (**C**) plasma creatinine, and (**D**) plasma indoxyl sulfate levels exhibited significant elevations following SNx. * *p* < 0.05 vs. sham mice; *n* = 7–13 in each group. BUN, blood urea nitrogen; BSA, bovine serum albumin; G-CSF, granulocyte colony-stimulating factor; HI, unilateral hindlimb ischemic operation; 2/3 Nx, 2/3 nephrectomy; SNx, subtotal nephrectomy; UNx, uninephrectomy.

Mice were allocated into three groups, including sham operation (Sham), SNx, and SNx administrated with G-CSF (SNx + G-CSF) (Figure 1A). To confirm the effect of SNx, we measured serum BUN and creatinine levels and completed an index of renal function 7 weeks after the SNx and sham operation. The results disclosed that the BUN level was notably elevated in mice that underwent SNx compared to the sham operation (Figure 1B,C). The plasma level of IS, a representative protein-bound uremic toxin, was remarkably greater in the SNx mice than the sham mice (Figure 1D).

### 3.2. G-CSF Reinstated Blood Flow Reperfusion in Ischemic Hindlimbs, Mobilized EPCs, and Elevated Plasma IL-10 Levels in SNx Mice

**Figure 2:**
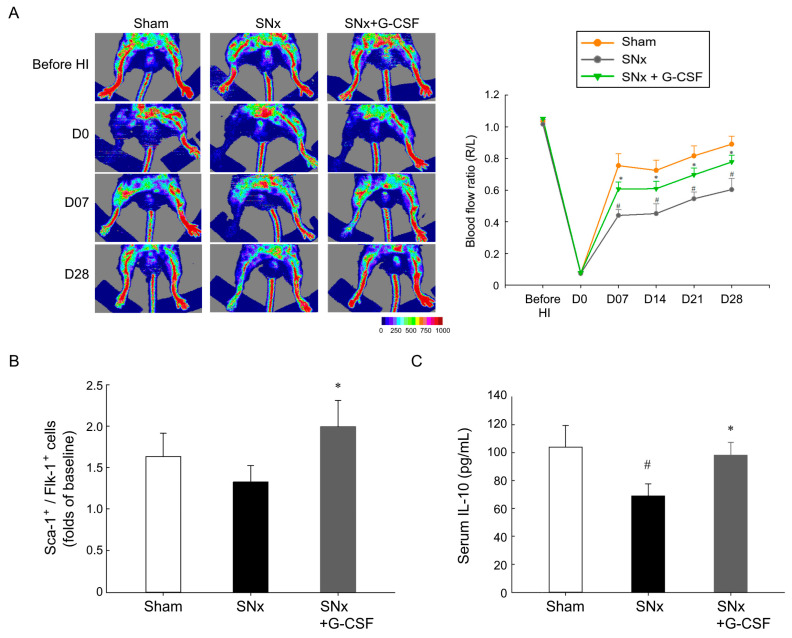
G-CSF improved blood reperfusion, EPC mobilization, and serum IL-10 levels in experiment mice. (**A**) Blood perfusion in ischemic hindlimbs was assessed using laser Doppler before, immediately after, and at 1, 2, 3, and 4 weeks post-hindlimb ischemia surgery. Quantitative analysis of blood perfusion was performed comparing ischemic limbs with control limbs (R/L). (**B**) Circulating Sca-1^+^/Flk-1^+^ cells were assessed via flow cytometry at baseline and 2 days following hindlimb ischemia surgery. Adjusted with baseline, G-GSF significantly increased Sca-1^+^/Flk-1^+^ cells at 2 days after HI as compared with the SNx group. (**C**) Serum IL-10 level markedly decreased after SNx. and G-CSF administration significantly reversed IL-10 level decreasing in SNx mice. ^#^ *p* < 0.05 vs. sham mice, * *p* < 0.05 vs. SNx mice; *n* = 7–13 in each group. EPC, endothelial progenitor cell; G-CSF, granulocyte colony-stimulating factor; IL-10, interleukin 10; HI, hindlimb ischemia surgery; SNx, subtotal nephrectomy; R, right; L, left.

Laser Doppler imaging was employed to measure the hindlimb ratio of blood flow in both ischemic and non-ischemic limbs before and at various time points following the HI procedure (Figure 2A). All three groups exhibited rapid declines in blood flow ratio subsequent to the HI procedure. The sham group displayed a ratio of approximately 0.9 after four weeks, while the SNx group demonstrated a considerably slower recovery, reaching a ratio of around 0.5 at the four-week mark. In contrast, the SNx + G-CSF group achieved a ratio of approximately 0.8 after four weeks. The above findings indicate that G-CSF mitigated the impact of experimental CKD on neovascularization.

We investigated whether EPC mobilization played a role in the protective effect of G-CSF in our CKD mice model. To assess this, we assessed the levels of Sca-1^+^/Flk-1^+^ cells at baseline and two days following the hindlimb ischemia procedure. The results revealed that the G-CSF group exhibited significantly higher levels of EPCs compared to the sham mice and SNx mice, but no significant difference was observed between the sham mice and SNx mice (Figure 2B). Since a previous study showed G-CSF can increase IL-10 level [13], we measured the serum IL-10 levels in experimental mice. The results indicated that G-CSF increased the serum IL-10 levels in the SNx groups (Figure 2C). Based on the above findings, we further investigated whether G-CSF could elevate the expression of IL-10 in muscles from ischemic hindlimbs in SNx mice.

### 3.3. G-CSF Restored Impaired Neovascularization Induced by Ischemia in SNx Mice

**Figure 3:**
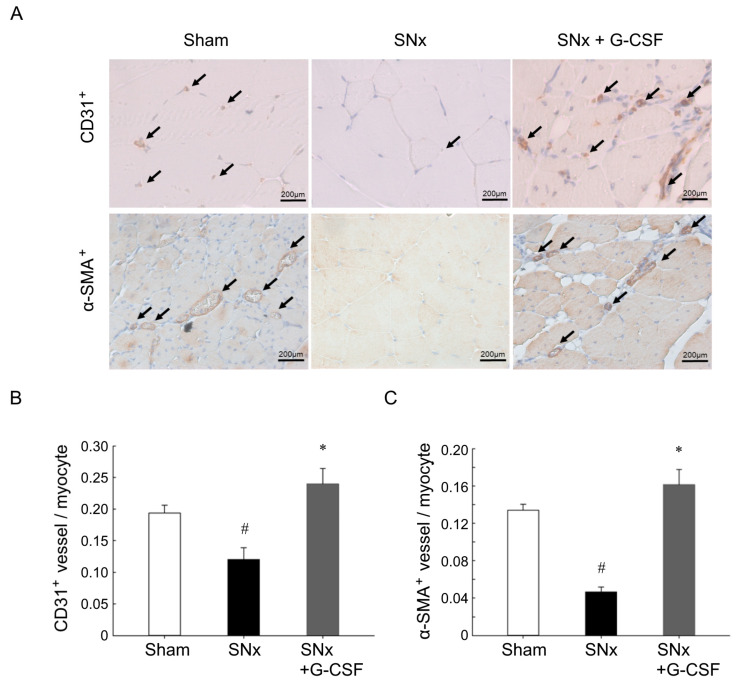
G-CSF increased neovascularization in ischemic limbs in SNx mice. (**A**) CD31^+^ capillaries and α-SMA^+^ small arteries were identified through co-staining for CD31^+^ (brown) and α-SMA^+^ (brown), with hematoxylin (blue) used as a counterstain in the ischemic muscles of the three study groups, four weeks after hindlimb ischemia surgery. The quantification included (**B**) CD31^+^ capillaries and (**C**) α-SMA^+^ cells within the ischemic muscles. ^#^ *p* < 0.05 vs. Sham mice; * *p* < 0.05 vs. SNx mice; *n* = 7–13 in each group. G-CSF, granulocyte colony-stimulating factor; SNx, subtotal nephrectomy.

To evaluate the impact of G-CSF on neovascularization, we examined the density of blood vessels in the ischemic hindlimbs four weeks after the ischemic surgery. The findings disclosed that the intensity of CD31^+^ capillaries (Figure 3A,B) and α-SMA^+^ small arteries (Figure 3A,C) were notably higher in the ischemic hindlimbs of the sham-operated group in comparison to the SNx group. Furthermore, G-CSF therapy successfully counteracted the impact of the SNx procedure.

### 3.4. G-CSF Modulates Protein Expression in Ischemic Hindlimbs from SNx Mice

**Figure 4:**
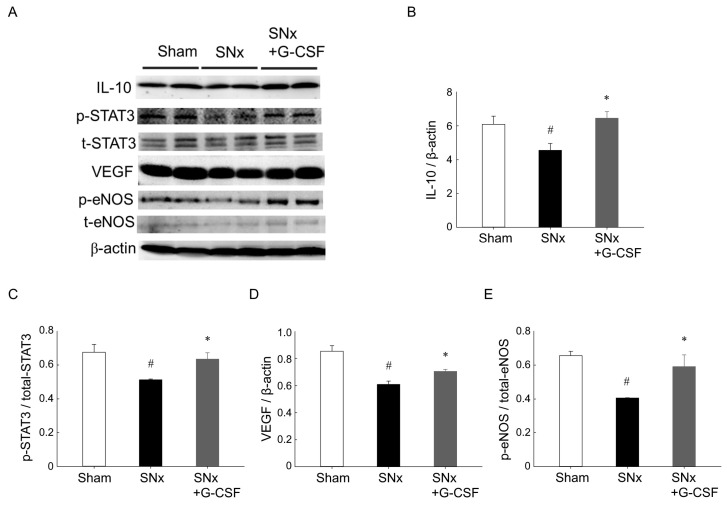
Protein expressions from ischemic limbs. Muscle tissue samples were collected from the ischemic limbs of the three study groups two weeks post-hindlimb ischemia surgery. (**A**) Western blotting was used to verify the presence of IL-10, p-STAT3, STAT3, VEGF, p-eNOS, and eNOS proteins. (**B**–**E**) Quantification was performed to assess the protein expressions of p-eNOS, eNOS, IL-10, p-STAT3, STAT3, and VEGF in the ischemic limbs. ^#^ *p* < 0.05 vs. Sham mice; * *p* < 0.05 vs. SNx mice; *n* = 4 in each group. eNOS, endothelial nitric oxide synthase; G-CSF, granulocyte colony-stimulating factor; IL, interleukin; p, phosphate; SNx, subtotal nephrectomy; STAT, signal transducer and activator of transcription; VEGF, vascular endothelial growth factor.

At the four-week mark following the hindlimb ischemic surgery, we employed western blotting to quantify the levels of p-eNOS, IL-10, p-STAT3, and VEGF in the muscles of the ischemic limbs (Figure 4). Our findings demonstrated a notable decrease in the levels of p-eNOS, IL-10, p-STAT3, and VEGF expression in SNx mice compared to sham-operated mice. However, the administration of G-CSF effectively reversed these effects in SNx mice.

### 3.5. G-CSF Modulates Protein Expression in Ischemic Hindlimbs from SNx Mice

**Figure 5:**
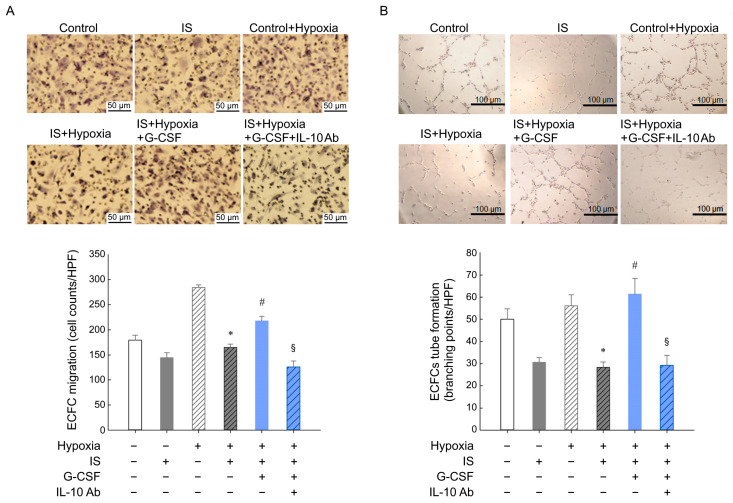
G-CSF reversed indoxyl-sulfate-inhibited migration and tube formation of human endothelial colony-forming cells. (**A**) Following a 24 h pretreatment with indoxyl sulfate, ECFCs were cultured in a Boyden chamber under hypoxic conditions (1% oxygen) for an additional 24 h. Subsequently, the cells were stained with hematoxylin. (**B**) The average total number of migrated cells was quantified using computer software. * *p* < 0.05 vs. hypoxia cells; ^#^ *p* < 0.05 vs. hypoxia with indoxyl sulfate; ^§^ *p* < 0.05 vs. hypoxia with indoxyl sulfate and G-CSF, *n* = 4 in each group. ECFC, endothelial colony forming cell; G-CSF, granulocyte colony-stimulating factor; IS, indoxyl sulfate; IL, interleukin; VEGF, vascular endothelial growth factor.

**Figure 6:**
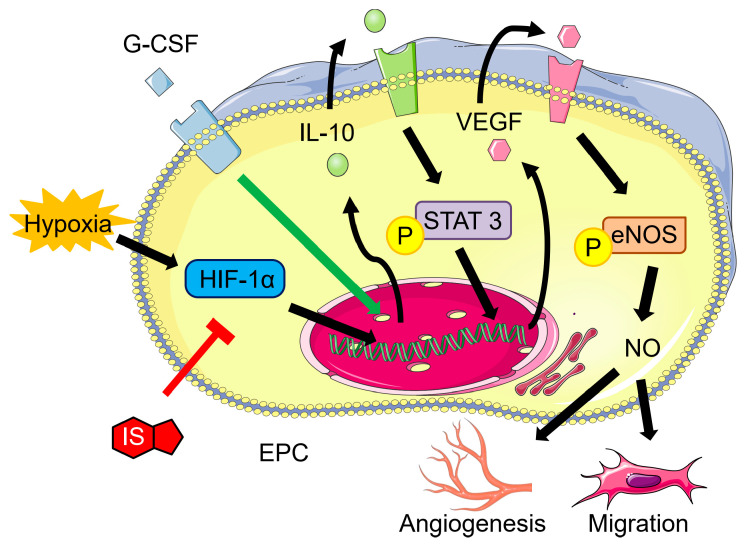
Schematic representation of the protective effect of G-CSF and its improvement of endothelial progenitor cell-mediated neovascularization. Administration of G-CSF ameliorates IS-impaired angiogenesis via the IL-10/STAT3/VEGF pathway in human ECFCs. The results were mutually supported in mice that underwent subtotal nephrectomy.

In our study, we employed the MTT assay to examine how G-CSF affects ECFCs, as presented in supplementary Figure S1 (Supplementary Materials). Our findings revealed that cells treated with 45, 450, or 4500 pg/mL of G-CSF demonstrated comparable viability to the control. However, the viability of cells treated with 45,000 pg/mL G-CSF was notably lower. As a result, we opted to utilize 450 pg/mL of G-CSF for subsequent experiments involving ECFCs. Angiogenesis involves various processes such as enhanced migration, invasion, and subsequent differentiation of endothelial cells into capillaries. Therefore, we aimed to evaluate the impact of G-CSF on ECFC angiogenesis using an in vitro assay that simulates the in vivo response of ECFC migration and tubule formation. The results revealed that the hypoxia group exhibited greater cell migration compared to the control group (normoxia). Additionally, IS, which is believed to contribute to reduced angiogenesis in CKD, diminished cell migration in the hypoxia group. However, the introduction of 450 pg/mL G-CSF effectively reversed the inhibitory effect caused by IS (Figure 5A). Similarly, IS was found to suppress tube formation, but this effect was reversed with the addition of 450 pg/mL G-CSF (Figure 5B). Nevertheless, the protective effect of G-CSF was nullified when IL-10 Ab was administered (Figure 5). Figure 6 presents a schematic illustration depicting the proposed mechanism through which G-CSF enhances endothelial progenitor cell-mediated neovascularization. Administration of G-CSF ameliorates IS-impaired angiogenesis via the IL-10, STAT3, and VEGF signaling pathway in ECFCs. The results were mutually supported in mice that underwent subtotal nephrectomy.

## 4. Discussion

Peripheral arterial disease poses a significant clinical challenge for patients with CKD. Lifestyle modifications, such as regular exercise, smoking cessation, and weight management, are among the strategies that can assist in managing PAD in these patients. Additionally, medications like statins, antiplatelet agents, and angiotensin-converting enzyme inhibitors may be employed to manage PAD and decrease the risk of cardiovascular events [19]. In certain cases, revascularization procedures, such as angioplasty and stenting, may be necessary to restore blood flow to the affected area [20]. However, the success rates of these interventions may be lower in ESRD patients. Reducing risk factors, including hypertension, hyperlipidemia, and hyperglycemia, can prevent and provide relief from PAD. However, patients with CKD encounter a distinct challenge as indoxyl sulfate (IS), a uremic toxin, poses an important risk factor for PAD [21]. Our previous study has shown that IS impairs neovascularization by hindering EPC mobilization and angiogenesis [9]. Nevertheless, administration of AST-120, which lowers serum IS, significantly improved ischemic hindlimb neovascularization in a subtotal nephrectomy animal model. To manage PAD in patients with CKD, a multidisciplinary approach is required to address their unique challenges. Promoting angiogenesis emerges as a promising therapeutic avenue for PAD, particularly among patients with CKD.

This present study shows that G-CSF reverses IS-induced inhibition of angiogenesis. We also examined the mechanism underlying this effect. G-CSF is a well-known myeloid growth factor, and previous studies have assessed the use of G-CSF as a prophylactic measure during chemotherapy-induced neutropenia, for retreatment after prior cycles of neutropenic fever, and to reduce the duration of chemotherapy-related neutropenia in afebrile patients [22]. Due to these successful clinical applications of G-CSF, we expect that it may also be effective for treatment of other conditions. Some studies demonstrated that G-CSF can increase angiogenesis, and other studies demonstrated that G-CSF suppresses immune reactions by upregulating IL-10 [13]. These previous studies motivated us to evaluate the protective effect of G-CSF on PAD in the presence of CKD. To the best of our knowledge, this study is the first to demonstrate the impact of G-CSF on PAD in the context of a CKD animal model.

Neovascularization is triggered by oxygen deficiency, prompting ischemic cells to release pro-angiogenic factors that attract nearby endothelial cells and stimulate cell proliferation [23,24]. These factors also support the integration of circulating EPCs into the local endothelial cell population. Mobilizing EPCs from the bone marrow is a promising strategy for enhancing postnatal neovascularization. Stromal cell–derived factor-1 (SDF-1) and VEGF, mainly produced in ischemic areas, play key roles in EPC mobilization [25,26,27,28]. Elevating circulating VEGF levels through methods like recombinant protein therapy or plasmid administration expands the pool of circulating EPCs in both experimental and clinical settings [29,30]. Additionally, virus-mediated gene delivery of angiopoietin-1 or SDF-1 increases EPC levels in animal models [30,31]. Some cytokines, such as G-CSF or granulocyte monocyte colony-stimulating factor (GM-CSF), are also relevant. G-CSF, a long-standing clinical tool for BMT, can mobilize hematopoietic stem and progenitor cells and increase the levels of progenitor cells in the peripheral blood [32,33].

Our study showed that G-CSF significantly restored blood reperfusion after HI surgery, and there was no statistically significant disparity in hindlimb blood flow observed between the SNx + G-CSF group and the sham surgery group. These findings confirm that G-CSF promotes angiogenesis in the presence of CKD. As G-CSF is recognized for its ability to induce cell mobilization, we additionally quantified the number of circulating EPCs following HI surgery. The data demonstrate a significant increase in the number of EPCs in the peripheral blood at two days following the HI surgery with the administration of G-CSF.

Ischemia upregulates HIF-1α, which then induces VEGF, leading to increased angiogenesis. IL-10 is also a pro-angiogenic factor, and previous research indicated that it promotes VEGF by increasing STAT-3 phosphorylation [34,35]. These findings support our hypothesis that G-CSF increases IL-10 expression, as well as the downstream molecules p-STAT3 and VEGF. eNOS, an enzyme upregulated by VEGF-mediated phosphorylation, converts L-arginine to L-citrulline while producing NO, thereby promoting cell proliferation, migration, and tubule formation. Our findings suggest that G-CSF administration led to a significant increase in the level of p-eNOS in ECFCs. We further investigated the impact of G-CSF on EPC angiogenesis using trans-well migration and tube formation assays, which mimic the migration of circulating EPCs towards ischemic tissue and the formation of capillary networks in vivo. Our study results showed that G-CSF increased ECFC migration under hypoxia, and that IS interfered with this effect.

In conclusion, our study utilizing a mouse model of neovascularization in the presence of CKD demonstrates elevated IS levels following subtotal nephrectomy, leading to impaired reperfusion of the ischemic limb. The administration of G-CSF resulted in an increased number of circulating EPCs in the peripheral blood and promoted signaling of the IL-10/p-STAT-3/VEGF/p-eNOS pathway in the ischemic limb. G-CSF also promoted angiogenesis of ECFCs. Consequently, this experiment’s originality and novelty stem from the utilization of this animal model, which furnishes evidence indicating that G-CSF has the potential to serve as a promising therapeutic agent for treating PAD in patients with CKD. Since our study has demonstrated the effectiveness of this treatment in animals, in the future, further human trials will contribute to the results of this foundational research.

## Data Availability

The data used to support the findings of this study are available from the corresponding author upon request.

## Supplementary Materials

The following supporting information can be downloaded at: https://www.mdpi.com/article/10.3390/pharmaceutics15102380/s1, Figure S1: Cell viability of ECFCs 24 h after culture with various concentrations of G-CSF determined with a 3-(4,5-dimethylthiazol-2-yl)-2,5-diphenyl tetrazolium bromide assay.
