# Supplementary material for: Granulocyte Colony-Stimulating Factor Improves Endothelial Progenitor Cell-Mediated Neovascularization in Mice with Chronic Kidney Disease
Source: Pharmaceutics. 2023 Sep 25;15(10):2380. doi: 10.3390/pharmaceutics15102380 (PMC10610103; doi:10.3390/pharmaceutics15102380)
Supplement: Supplementary file 1 [file pharmaceutics-15-02380-s001.zip › pharmaceutics-2586137-supplementary.pdf]

Supplementary Material

S1 Supplementary Methods

S1.1 3-(4,5-Dimethylthiazol-2-yl)-2,5-diphenyl tetrazolium bromide assay for cell viability

3-(4,5-Dimethylthiazol-2-yl)-2,5-diphenyl tetrazolium bromide (MTT) assay (Sigma Chemical Co., St. Louis, MO, USA) was used to measure cell viability. The principle of this assay is that mitochondrial dehydrogenase in viable cells reduces MTT to a blue formazan. Briefly, cells were grown in 96-well plates and incubated with various concentrations of G-CSF for 24 h. After washing human endothelial colony forming cells (ECFCs) with phosphate-buffered saline (PBS), 100 μL of medium containing MTT (0.5 mg/mL) was added to each well for incubation at 37°C for 4 h. The medium was then carefully removed to avoid disturbing the formazan crystals that had formed. One hundred microliters of dimethyl sulfoxide, which solubilizes formazan crystals, was added to each well, and the absorbance of the solubilized blue formazan was read at 540 nm using a microplate reader (Multiskan Ex, Thermo Labsystems, Beverly, MA). Dimethyl sulfoxide was the blank. The reduction in optical density caused by the drug was used as a measurement of cell viability. Optical density in each IS-treated group was normalized to the optical density of the cells incubated in the control medium, which were considered 100% viable (the untreated group).

S2 Supplementary Figures

S2.1 Supplementary Figure S1

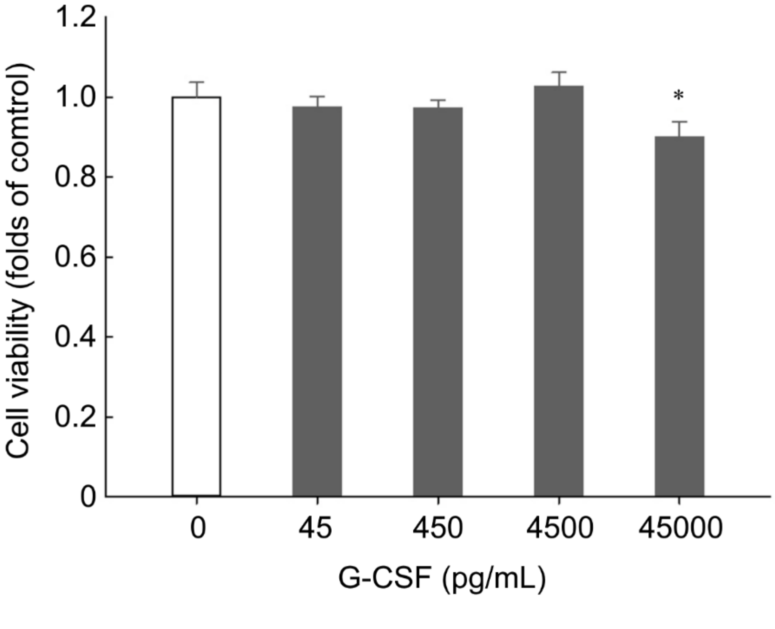

Supplementary Figure S1. Cell viability of ECFCs 24 h after culture with various concentrations of G-CSF determined with 3-(4,5-dimethylthiazol-2-yl)-2,5-diphenyl tetrazolium bromide assay. The percentage of cell viability in the indoxyl sulfate–treated groups was compared with that in the untreated group (cell viability = 100%). The data are expressed as the mean ± SEM from 3 independent experiments.
